# TRICK or TRP? What Trpc2^−/−^ mice tell us about vomeronasal organ mediated innate behaviors

**DOI:** 10.3389/fnins.2015.00221

**Published:** 2015-06-23

**Authors:** C. Ron Yu

**Affiliations:** ^1^Stowers Institute for Medical ResearchKansas City, MO, USA; ^2^Department of Anatomy and Cell Biology, University of Kansas Medical CenterKansas City, KS, USA

**Keywords:** vomeronasal organ, Trpc2, signaling pathways, mating behavior, aggressive behavior, neomorphic behaviors

## Abstract

The vomeronasal organ (VNO) plays an important role in mediating semiochemical communications and social behaviors in terrestrial species. Genetic knockout of individual components in the signaling pathways has been used to probe vomeronasal functions, and has provided much insights into how the VNO orchestrates innate behaviors. However, all data do not agree. In particular, knocking out Trpc2, a member of the TRP family of non-selective cationic channel thought to be the main transduction channel in the VNO, results in a number of fascinating behavioral phenotypes that have not been observed in other animals whose vomeronasal function is disrupted. Recent studies have identified signaling pathways that operate in parallel of Trpc2, raising the possibility that Trpc2 mutant animals may display neomorphic behaviors. In this article, I provide a critical analysis of emerging evidence to reconcile the discrepancies and discuss their implications.

In terrestrial vertebrates, endocrine changes, and stereotypic innate behaviors are often triggered by pheromones. The mammalian vomeronasal organ (VNO) plays an important role in orchestrating pheromone-mediated behaviors (Eisthen and Wyatt, 2006; Tirindelli et al., 2009). In early studies, the functional role of VNO has been derived from ablation experiments in which the VNO is surgically disrupted (VNX) (Bean, 1982; Clancy et al., 1984; Beauchamp et al., 1985; Lepri et al., 1985; Maruniak et al., 1986; Lepri and Wysocki, 1987; Bean and Wysocki, 1989; Labov and Wysocki, 1989; Wysocki and Lepri, 1991; Wysocki et al., 2004). The advent of molecular biology made it possible to genetically manipulate individual components in VNO signaling pathways and provide insights into the mechanisms of VNO mediated behaviors (Del Punta et al., 2002; Leypold et al., 2002; Stowers et al., 2002; Norlin et al., 2003; Kelliher et al., 2006; Kimchi et al., 2007; Chamero et al., 2011; Kim et al., 2012; Leinders-Zufall et al., 2014; Oboti et al., 2014). A consensus that emerges from these studies is that the VNO is essential in triggering territorial aggression. In line with surgical ablation experiments, removing any component of the VNO signaling pathway, including vomeronasal receptors, G proteins, or ion channels, results in diminished aggression in mice (Bean, 1982; Clancy et al., 1984; Maruniak et al., 1986; Bean and Wysocki, 1989; Labov and Wysocki, 1989; Del Punta et al., 2002; Leypold et al., 2002; Stowers et al., 2002; Norlin et al., 2003; Kimchi et al., 2007; Chamero et al., 2011; Kim et al., 2012; Oboti et al., 2014). Genetic mutations that affect VNO function also lead to loss of avoidance to predator or sick animals (Papes et al., 2010; Boillat et al., 2015).

The data on mating behaviors, especially the mounting behaviors displayed by male animals, are less consistent. One of the most interesting behavioral observations comes from mice with knock out mutation of Trpc2, a member of the TRP superfamily of ion channels (Liman et al., 1999). Although several TRP members have been detected in the VNO (Zufall, 2014), Trpc2 appears to be the only one expressed in the vomeronasal sensory neurons (VSNs) as verified by *in situ* hybridization, immunofluorescent staining and electron microscopy (Liman et al., 1999; Menco et al., 2001; Leypold et al., 2002). While Trpc2^−/−^ males display normal mounting behaviors toward female mice, they also indiscriminately mount intruder males (Leypold et al., 2002; Stowers et al., 2002). Most strikingly, female Trpc2^−/−^ mice exhibit hallmarks of male mating behaviors, including solicitation, mounting, and pelvic thrust, toward female and male mice alike (Kimchi et al., 2007). The behavioral phenotypes of Trpc2^−/−^ mice do not recapitulate those observed in VNX rodents (Powers and Winans, 1975; Winans and Powers, 1977; Clancy et al., 1984; Meredith, 1986; Saito and Moltz, 1986; Lepri and Wysocki, 1987; Wysocki and Lepri, 1991; Pfeiffer and Johnston, 1994; Kolunie and Stern, 1995).

In the conventional model of VNO function, male mounting behavior is triggered by pheromone stimulation, through what is considered as the releasing effect of pheromones (Vandenbergh, 1983). Based on the observations from the Trp2^−/−^ mice, Dulac and colleagues proposed an alternative model of VNO function (Stowers et al., 2002). In this new model, mounting is the default behavior triggered by non-VNO sensory input. The function of the VNO is to “ensure gender specific behavior,” which inhibits a male mouse from mounting a male (Stowers et al., 2002).

The new interpretation of VNO function is controversial and the discrepancies in behavioral data raise important questions about the functional role of VNO in innate behaviors. At the center of this controversy are two important questions: what is the role played by Trpc2 in pheromone sensing? And is mounting a default behavior that does not require VNO activation? Here I evaluate recent development in the field and attempt to reconcile differences in the experimental results.

## Have Trpc2^−/−^ mice lost VNO function specifically and completely?

Two groups generated the Trpc2^−/−^ mice independently and reported the loss of territorial aggression and the display of male-male mounting behaviors (Leypold et al., 2002; Stowers et al., 2002). However, they disagreed on whether Trpc2^−/−^ animals completely lost pheromone induced responses. Whereas Stowers and colleagues reported a complete loss of pheromone-triggered activities, residual responses were observed in the studies of Leypold et al. Indeed, Leypold and colleagues cautioned that the residual response might affect how the behavioral data was interpreted.

Since the publication of the initial Trpc2^−/−^ papers, new evidence has emerged from electrophysiological studies challenging the notion that Trpc2 mutation resulted a “null” VNO. Liman first discovered a calcium-activated non-selective (CaNS) cationic channel in hamster VNO neurons (Liman, 2003). A similar conductance was later reported in mouse (Spehr et al., 2009). Although the identity of the channel remains unknown to date, these studies provide the first evidence of Trpc2 independent activation of VNO neurons.

Recently a comprehensive picture of VNO signaling has emerged from the studies by several groups. Delay and colleagues described calcium-activated BK and calcium-activated chloride channel (CACC) in mouse VNO (Zhang et al., 2008; Yang and Delay, 2010). My group later demonstrated that pheromone triggered CACC current was present in VNO neurons of the Trpc2^−/−^ mice (Kim et al., 2011). The CACC now has been identified as TMEM16A/anoctamin1 (Amjad et al., 2015). Delay and colleagues also identified an arachidonic acid dependent signaling pathway in VNO of the Trpc2^−/−^ mouse, with a different knockout line of Trpc2 (Zhang et al., 2010).

**Figure 1:**
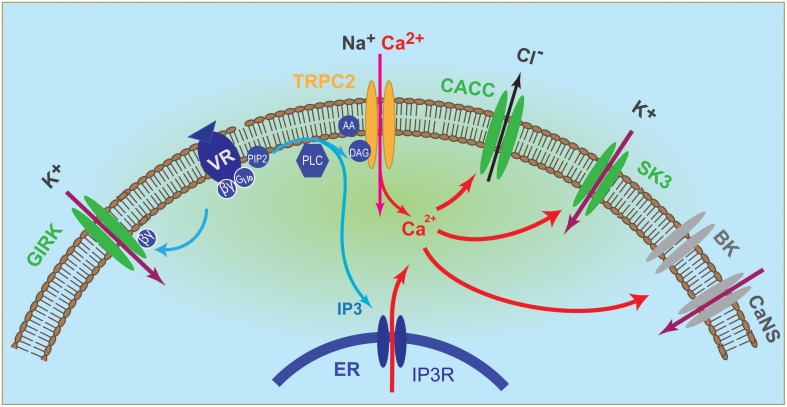
**Illustration of vomeronasal neuron signaling pathway**. Binding of ligands to their cognate receptors trigger the activation of Gαi2/Gαo, which in turn activate the phospholipase C (PLC) to produce inositol- 1, 4, 5 - triphosphate (IP3) and diacylglycerol (DAG). DAG activates Trpc2 channel, leading to influx of cationic ions, including Ca^2+^, whereas IP3 triggers release of Ca^2+^ from intracellular stores. Elevated intracellular Ca^2+^ in turn activates calcium-activated chloride conductance (CACC) and the small conductance calcium-activated potassium channel SK3. Activation of G protein also releases βγ subunits, which activate the G-protein activated inward rectifier channel (GIRK). Both GIRK and SK3 mediate influx of potassium to depolarize the VSN because of a high extracellular [K^+^] in the vomeronasal lumenal mucus. Elevated Ca^2+^ can also activates the large conductance calcium-activated potassium channel BK and an unidentified calcium-activated non-selective (CaNS) cationic channel. These two conductance may reside in the dendrite or in the cell body.

In addition, calcium-activated small conductance potassium channel SK3 and G-protein activated inward rectifier potassium channel GIRK were found to act as primary conductance channel in the VSN dendrite and acted in parallel of Trpc2 (Kim et al., 2012). Importantly, the two K channels were depolarizing *in vivo* due to the unusually high K^+^ concentrations in the VNO lumen (Kim et al., 2012). Changes in this ionic environment can regulate VNO responses by altering the reversal potential of K^+^, and it remains to be determined whether conditions such as strain, age, and hormonal status can influence K^+^ homeostasis in the lumen. These discoveries have led to a revised version of the signaling pathways in the VNO that include at least four ion channels directly activated by pheromone stimulation (Figure 1). Pheromones can trigger CACC, SK3, and GIRK independent of Trpc2, although Ca^2+^ entry through Trpc2 can augment CACC and SK3 activation. Trpc2 channel accounts for ~30–40% of the total excitation and Trpc2^−/−^ neurons retain substantial response to pheromones (Kim et al., 2012).

Electrophysiological evidence of Trpc2-independent activation of VNO are supported by histology and behavior analyses. Hasen and Gammie reported that the medial amygdala, which primarily received input from the VNO, was strongly activated in Trp2^−/−^ mice by soiled bedding (Hasen and Gammie, 2009, 2011). Zufall and colleagues found that Bruce effect, pregnancy block induced by strange males, was intact in Trpc2^−/−^ but not VNX mice (Kelliher et al., 2006).

The impact of Trpc2 mutation on pheromone signaling is likely not uniform. An important observation of Trpc2^−/−^ VNO was a significant loss of basal layer neurons (Stowers et al., 2002; Kim et al., 2012), which expressed Gαo and the V2r family of receptors (Herrada and Dulac, 1997; Matsunami and Buck, 1997; Ryba and Tirindelli, 1997). Unfortunately, the data were buried in supplemental materials and did not garner the attention they deserved (Stowers et al., 2002; Kim et al., 2012). The study by Hasen and Gammie, on the other hand, clearly showed pronounced reduction of the posterior accessory olfactory bulb in Trpc2^−/−^ mice (Hasen and Gammie, 2009). Thus, it is possible that the activation of basal VSNs is more severely affected than those in the apical layer. This difference may have important implications in behaviors (see below) and explain the apparent loss of VNO activation in the Stowers et al. study. In this study, the major difference in VSN activity between control and Trpc2^−/−^ mice were recorded by laying the sensory epithelium face up on top of an electrode array with the electrodes preferentially made contact with the basal VSNs (Stowers et al., 2002). If Trpc2^−/−^ have a more severe impact on the basal cells, this recording configuration may report diminished activity. Activity in the apical layer, which is less affected, may be occluded from recording by the remaining basal cells.

Does Trpc2 mutation affect the VNO specifically? Trpc2 was initially thought to be exclusively expressed in VNO. Recent evidence suggests that Trpc2 is expressed in a subset of MOE neurons, embryonic brain tissues, and non-neuronal cells, raising the question whether Trpc2^−/−^ affects VNO function specifically (Elg et al., 2007; Boisseau et al., 2009; Hirschler-Laszkiewicz et al., 2012; Omura and Mombaerts, 2014, 2015). In an elegant study, Mombaerts and colleagues knocked in the lacZ gene into the Trpc2 locus and traced the projections of Trpc2-expressing neurons. They discovered that Trpc2 was expressed by two types of MOE neurons projecting to specific glomeruli in ventral side of the main olfactory bulb (Omura and Mombaerts, 2014, 2015). These findings suggest that Trpc2 may carry additional functions in the main olfactory system, as well as other brain areas, and the behavioral phenotypes observed in Trpc2^−/−^ mice are unlikely to be the sole results of VNO disruption.

## Is mounting behavior dependent on a functional VNO?

VNO ablation experiments, performed by a number of labs over several decades, have consistently shown that VNX rodents exhibit diminished mating behaviors (Powers and Winans, 1975; Winans and Powers, 1977; Clancy et al., 1984; Meredith, 1986; Saito and Moltz, 1986; Lepri and Wysocki, 1987; Wysocki and Lepri, 1991; Pfeiffer and Johnston, 1994; Kolunie and Stern, 1995). Trpc2^−/−^ males, on the other hand, show indiscriminate mounting toward intruders (Leypold et al., 2002; Stowers et al., 2002). The most striking observation is that Trpc2^−/−^ females also display mounting behaviors (Kimchi et al., 2007). These behavior phenotypes are rarely observed in wildtype animals. In an attempt to explain the discrepancy in the results, Kimchi and colleagues suggested that VNX surgery could inadvertently cause blood clog in the nasal passage and block odor entry (Kimchi et al., 2007). This scenario is unlikely because mice are obligate nasal breathers. Indeed, several studies have shown that VNX animals display normal approach and investigation of odor sources, indicating that the animals can smell normally [reviewed (Wysocki and Lepri, 1991)]. VNX mice also exhibit investigation of urine source, even though they no longer show preference for urine from the opposite sex (Pankevich et al., 2004, 2006). A careful study also failed to replicate some of the male-typical responses in VNX female mice in the Kimchi study (Martel and Baum, 2009).

In addition to VNX, chemical and genetic ablations of the MOE also lead to diminished investigation of the conspecifics, urine preference and mating behaviors (Thor and Flannelly, 1977; Bean, 1982; Kolunie and Stern, 1995; Keller et al., 2006). CNGA2 knockout mice, which are anosmic because of the loss of an essential component in the olfactory signal transduction pathway, are compromised in mating behaviors (Mandiyan et al., 2005). These observations suggest that attraction by urinary odors can bring the animals to investigate the sources and enable the direct physical contact with non-volatile pheromones by the VNO. Loss of MOE function leads to the loss of odor-evoked investigation and, in turn, could diminish pheromone detection by the VNO. These data should not be construed as definitive evidence that the MOE, but not the VNO, is required to trigger mounting.

Along with studies of VNX animals of several species, a number of transgenic mouse lines that have various deficiencies in VNO function have been studied. These lines include mice with deletion of a V1r receptor cluster, knockout mutations of signaling molecules Gαi2 and Gαo, and mutations of ion channels SK3 and GIRK1(Del Punta et al., 2002; Norlin et al., 2003; Chamero et al., 2011; Kim et al., 2012). None of these lines exhibit male-male mounting or male-like sexual behaviors in the females.

Whereas, the loss of function studies suggesting that the VNO is required to trigger mating behaviors, our recent data demonstrate that pheromones components are sufficient to trigger mating behavior (Haga-Yamanaka et al., 2014). We have previously shown that the VNO recognizes cues that signal the sex and reproductive status of the animal (He et al., 2008, 2010). We have recently identified two sets of pheromone cues (Haga-Yamanaka et al., 2014). A urinary fraction purified from female urine, which we call T16, contains sex-specific cues that signal the carrier as females. This fraction is recognized by a subset of the V1re clade of receptors. We also show that sulfated estrogens specifically activate the V1rj clade of receptors and signal estrus status of the female mice. These cues do not activate the MOE. Although neither sulfated estrogens nor T16 alone alters baseline mating toward ovariectomized females, combining the two cues together elicits strong mounting behaviors (Haga-Yamanaka et al., 2014).

The confluence of data, therefore, suggest that mounting is not a default behavioral output and the VNO is required to trigger this mating behaviors. The notions that non-VNO sensory cues conveying conspecific information to elicit mating as a default behavior is primarily derived from observations of Trpc2^−/−^ mice. This conclusion critically depends on the assumption that Trpc2^−/−^ causes a complete loss of VNO function. As the VNO retains partial function in Trpc2^−/−^mice, it is likely that aberration in VNO signaling in transmitting pheromone information causes aberrant mating behaviors. Indeed, male to male mounting exhibited by Trpc2^−/−^ mice is also observed in double mutant mice that also carry Cnga2^−/−^ or SK3^−/−^ alleles (Kim et al., 2012; Fraser and Shah, 2014).

## Neomorphic or displacement behaviors in Trpc2^−/−^ mutants?

What is the nature of the aberrant behaviors observed in Trpc2^−/−^ mice? Classically, the display of behaviors out of context is categorized as displacement activities (Tinbergen, 1989). Animals have a restricted repertoire of innate behaviors preprogrammed in the brain circuitry. Within the same animal, circuit mechanism exists to ensure that antagonistic behavioral patterns are displayed in a mutually exclusive fashion. Displacement reactions arise when there are motivational conflicts, frustration of consummatory acts or physical thwarting of performance (Tinbergen, 1989). Lorenz has described that when fighting drives are obstructed in cranes, they exhibit displacement preening (Lorenz, 1935). Trpc2^−/−^ males have the ability to fight when provoked in a neutral arena, yet they mount instead of attack intruder males (Leypold et al., 2002). Female Trpc2^−/−^ mice show diminished female-specific behaviors such as maternal aggression and lactation, but instead exhibit male-typical sexual behaviors (Leypold et al., 2002; Stowers et al., 2002; Kimchi et al., 2007). It is possible that pheromone signaling in Trpc2^−/−^ mice generate conflicting motivational drive, leading to the replacement of normal responses with an out-of-context substitute. However, no classical case of displacement activities involves a genetic mutation. Therefore, although one could add genetic changes as a cause of displacement activities, it will be more appropriate to characterize behaviors in Trpc2^−/−^ mice as neomorphic. Mating and aggression may be on the same continuum of a behavioral spectrum. The same set of neurons in the ventral medial hypothalamic nucleus drive either mating or aggression depending on the level of activation (Lee et al., 2014). Aberrant input from the VNO is likely to feed into this circuit and induce inappropriate display of mating or aggression.

What may cause neomorphic behaviors in the Trpc2^−/−^ mice? I present two hypotheses to stimulate discussion. The first concerns the development of the vomeronasal circuit, which is linked to gonadotropin releasing hormone (GnRH) neurons in the hypothalamus and preoptic area (Meredith, 1998). GnRH cells migrate along the vomeronasal projection to reach the brain (Schwanzel-Fukuda, 1999). It remains unknown how the development of vomeronasal neurons may affect this migration and the establishment of GnRH neuron connections. A substantial loss of the basal neurons may cause a mis-wiring of the mating/aggression circuit. In addition, physiological changes in Trpc2^−/−^ mice may impact circuit development. Both male and female Trpc2^−/−^ mice have higher testosterone levels than wildtypes (Leypold et al., 2002; Kimchi et al., 2007). As masculinization of the brain could result from elevated testosterone or estrogen levels in adults, as well as from estrogen treatment in neonatal pups (Paup et al., 1972; Baum, 2009; Martel and Baum, 2009; Wu et al., 2009), it is possible that deficiency in pheromone detection during development could lead to brain masculinization in females.

Second, Trp2^−/−^ may directly influence how pheromones are perceived. The basal layer, V2r-expressing VSNs that are lost in Trpc2^−/−^ mice detect polypeptide pheromones, some of which have been shown to elicit aggression (Chamero et al., 2007). ESP22, a peptide pheromone secreted by juvenile mice, has a powerful effect in inhibiting male mating behaviors (Ferrero et al., 2013). Loss of either Trpc2 or ESP22 leads to mounting of juveniles (Ferrero et al., 2013). It is possible that the loss of the basal layer cells in Trpc2^−/−^, compounded by the partial loss of sensitivity in the remaining neurons, weakens signals that inhibit mating and trigger aggression. The net effect could be the mis-interpretation of pheromone cues and a switch from aggression to mating. Finally, it remains possible that the loss of Trpc2 outside of VNO could contribute to neomorphic behaviors.

## Concluding remarks

Behaviors displayed by the Trpc2^−/−^ are fascinating. They capture the imagination of the public and the experts alike. It also has become a requirement to use these mice to demonstrate whether an innate behavior is dependent on VNO function. However, the impact of Trpc2^−/−^ on VNO function is more nuanced than previously thought. How Trpc2^−/−^ causes neomorphic behaviors remains largely unknown. As disruption of VNO function may influence both brain development and pheromone-triggered responses, more detailed studies are required to understand the physiological changes of Trpc2^−/−^ mice. It is important to use caution in using these mice to assess innate behaviors.

### Conflict of interest statement

The author declares that the research was conducted in the absence of any commercial or financial relationships that could be construed as a potential conflict of interest.

## References

[B1] Amjad A. Hernandez-Clavijo A. Pifferi S. Maurya D. K. Boccaccio A. Franzot J.. (2015). Conditional knockout of TMEM16A/anoctamin1 abolishes the calcium-activated chloride current in mouse vomeronasal sensory neurons. J. Gen. Physiol. 145, 285–301. 10.1085/jgp.201411348 25779870 PMC4380210

[B2] Baum M. J. (2009). Sexual differentiation of pheromone processing: links to male-typical mating behavior and partner preference. Horm. Behav. 55, 579–588. 10.1016/j.yhbeh.2009.02.008 19446074 PMC2684524

[B3] Bean N. J. Wysocki C. J. (1989). Vomeronasal organ removal and female mouse aggression: the role of experience. Physiol. Behav. 45, 875–882. 10.1016/0031-9384(89)90209-6 2780872

[B4] Bean N. J. (1982). Olfactory and vomeronasal mediation of ultrasonic vocalizations in male mice. Physiol. Behav. 28, 31–37. 10.1016/0031-9384(82)90097-X 7079320

[B5] Beauchamp G. K. Wysocki C. J. Wellington J. L. (1985). Extinction of response to urine odor as a consequence of vomeronasal organ removal in male guinea pigs. Behav. Neurosci. 99, 950–955. 10.1037/0735-7044.99.5.950 3843311

[B6] Boillat M. Challet L. Rossier D. Kan C. Carleton A. Rodriguez I. (2015). The vomeronasal system mediates sick conspecific avoidance. Curr. Biol. 25, 251–255. 10.1016/j.cub.2014.11.061 25578906

[B7] Boisseau S. Kunert-Keil C. Lucke S. Bouron A. (2009). Heterogeneous distribution of TRPC proteins in the embryonic cortex. Histochem. Cell Biol. 131, 355–363. 10.1007/s00418-008-0532-6 18989690

[B8] Chamero P. Katsoulidou V. Hendrix P. Bufe B. Roberts R. Matsunami H.. (2011). G protein G(alpha)o is essential for vomeronasal function and aggressive behavior in mice. Proc. Natl. Acad. Sci. U.S.A. 108, 12898–12903. 10.1073/pnas.1107770108 21768373 PMC3150917

[B9] Chamero P. Marton T. F. Logan D. W. Flanagan K. Cruz J. R. Saghatelian A.. (2007). Identification of protein pheromones that promote aggressive behaviour. Nature 450, 899–902. 10.1038/nature05997 18064011

[B10] Clancy A. N. Coquelin A. Macrides F. Gorski R. A. Noble E. P. (1984). Sexual behavior and aggression in male mice: involvement of the vomeronasal system. J. Neurosci. 4, 2222–2229. 6541245 10.1523/JNEUROSCI.04-09-02222.1984 PMC6564808

[B11] Del Punta K. Leinders-Zufall T. Rodriguez I. Jukam D. Wysocki C. J. Ogawa S.. (2002). Deficient pheromone responses in mice lacking a cluster of vomeronasal receptor genes. Nature 419, 70–74. 10.1038/nature00955 12214233

[B12] Eisthen H. L. Wyatt T. D. (2006). The vomeronasal system and pheromones. Curr. Biol. 16, R73–R74. 10.1016/j.cub.2006.01.038 16461264

[B13] Elg S. Marmigere F. Mattsson J. P. Ernfors P. (2007). Cellular subtype distribution and developmental regulation of TRPC channel members in the mouse dorsal root ganglion. J. Comp. Neurol. 503, 35–46. 10.1002/cne.21351 17480026

[B14] Ferrero D. M. Moeller L. M. Osakada T. Horio N. Li Q. Roy D. S.. (2013). A juvenile mouse pheromone inhibits sexual behaviour through the vomeronasal system. Nature 502, 368–371. 10.1038/nature12579 24089208 PMC3800207

[B15] Fraser E. J. Shah N. M. (2014). Complex chemosensory control of female reproductive behaviors. PLoS ONE 9:e90368. 10.1371/journal.pone.0090368 24587340 PMC3938725

[B16] Haga-Yamanaka S. Ma L. He J. Qiu Q. Lavis L. D. Looger L. L.. (2014). Integrated action of pheromone signals in promoting courtship behavior in male mice. eLife 3:e03025. 10.7554/eLife.03025 25073926 PMC4107909

[B17] Hasen N. S. Gammie S. C. (2009). Trpc2 gene impacts on maternal aggression, accessory olfactory bulb anatomy and brain activity. Genes Brain Behav. 8, 639–649. 10.1111/j.1601-183X.2009.00511.x 19799641 PMC2758541

[B18] Hasen N. S. Gammie S. C. (2011). Trpc2-deficient lactating mice exhibit altered brain and behavioral responses to bedding stimuli. Behav. Brain Res. 217, 347–353. 10.1016/j.bbr.2010.11.002 21070815 PMC3010422

[B19] He J. Ma L. Kim S. Nakai J. Yu C. R. (2008). Encoding gender and individual information in the mouse vomeronasal organ. Science 320, 535–538. 10.1126/science.1154476 18436787 PMC2602951

[B20] He J. Ma L. Kim S. Schwartz J. Santilli M. Wood C.. (2010). Distinct signals conveyed by pheromone concentrations to the mouse vomeronasal organ. J. Neurosci. 30, 7473–7483. 10.1523/JNEUROSCI.0825-10.2010 20519522 PMC2919682

[B21] Herrada G. Dulac C. (1997). A novel family of putative pheromone receptors in mammals with a topographically organized and sexually dimorphic distribution. Cell 90, 763–773. 10.1016/S0092-8674(00)80536-X 9288755

[B22] Hirschler-Laszkiewicz I. Zhang W. Keefer K. Conrad K. Tong Q. Chen S. J.. (2012). Trpc2 depletion protects red blood cells from oxidative stress-induced hemolysis. Exp. Hematol. 40, 71–83. 10.1016/j.exphem.2011.09.006 21924222 PMC3237850

[B23] Keller M. Douhard Q. Baum M. J. Bakker J. (2006). Destruction of the main olfactory epithelium reduces female sexual behavior and olfactory investigation in female mice. Chem. Senses 31, 315–323. 10.1093/chemse/bjj035 16484502 PMC2263131

[B24] Kelliher K. R. Spehr M. Li X. H. Zufall F. Leinders-Zufall T. (2006). Pheromonal recognition memory induced by TRPC2-independent vomeronasal sensing. Eur. J. Neurosci. 23, 3385–3390. 10.1111/j.1460-9568.2006.04866.x 16820028

[B25] Kim S. Ma L. Jensen K. L. Kim M. M. Bond C. T. Adelman J. P.. (2012). Paradoxical contribution of SK3 and GIRK channels to the activation of mouse vomeronasal organ. Nat. Neurosci. 15, 1236–1244. 10.1038/nn.3173 22842147 PMC3431453

[B26] Kim S. Ma L. Yu C. R. (2011). Requirement of calcium-activated chloride channels in the activation of mouse vomeronasal neurons. Nat. Commun. 2:365. 10.1038/ncomms1368 21694713 PMC3156823

[B27] Kimchi T. Xu J. Dulac C. (2007). A functional circuit underlying male sexual behaviour in the female mouse brain. Nature 448, 1009–1014. 10.1038/nature06089 17676034

[B28] Kolunie J. M. Stern J. M. (1995). Maternal aggression in rats: effects of olfactory bulbectomy, ZnSO4-induced anosmia, and vomeronasal organ removal. Horm. Behav. 29, 492–518. 10.1006/hbeh.1995.1285 8748510

[B29] Labov J. B. Wysocki C. J. (1989). Vomeronasal organ and social factors affect urine marking by male mice. Physiol. Behav. 45, 443–447. 10.1016/0031-9384(89)90153-4 2756033

[B30] Lee H. Kim D. W. Remedios R. Anthony T. E. Chang A. Madisen L.. (2014). Scalable control of mounting and attack by Esr1+ neurons in the ventromedial hypothalamus. Nature 509, 627–632. 10.1038/nature13169 24739975 PMC4098836

[B31] Leinders-Zufall T. Ishii T. Chamero P. Hendrix P. Oboti L. Schmid A.. (2014). A family of nonclassical class I MHC genes contributes to ultrasensitive chemodetection by mouse vomeronasal sensory neurons. J. Neurosci. 34, 5121–5133. 10.1523/JNEUROSCI.0186-14.2014 24719092 PMC4050176

[B32] Lepri J. J. Wysocki C. J. (1987). Removal of the vomeronasal organ disrupts the activation of reproduction in female voles. Physiol. Behav. 40, 349–355. 10.1016/0031-9384(87)90058-8 3310053

[B33] Lepri J. J. Wysocki C. J. Vandenbergh J. G. (1985). Mouse vomeronasal organ: effects on chemosignal production and maternal behavior. Physiol. Behav. 35, 809–814. 10.1016/0031-9384(85)90416-0 4080845

[B34] Leypold B. G. Yu C. R. Leinders-Zufall T. Kim M. M. Zufall F. Axel R. (2002). Altered sexual and social behaviors in trp2 mutant mice. Proc. Natl. Acad. Sci. U.S.A. 99, 6376–6381. 10.1073/pnas.082127599 11972034 PMC122956

[B35] Liman E. R. Corey D. P. Dulac C. (1999). TRP2: a candidate transduction channel for mammalian pheromone sensory signaling. Proc. Natl. Acad. Sci. U.S.A. 96, 5791–5796. 10.1073/pnas.96.10.5791 10318963 PMC21939

[B36] Liman E. R. (2003). Regulation by voltage and adenine nucleotides of a Ca2+-activated cation channel from hamster vomeronasal sensory neurons. J. Physiol. 548, 777–787. 10.1113/jphysiol.2002.037119 12640014 PMC2342889

[B37] Lorenz K. (1935). Der Kumpan in der Umwelt des Vogels. J. Ornithol. 83, 289–413. 10.1007/BF01905572

[B38] Mandiyan V. S. Coats J. K. Shah N. M. (2005). Deficits in sexual and aggressive behaviors in Cnga2 mutant mice. Nat. Neurosci. 8, 1660–1662. 10.1038/nn1589 16261133

[B39] Martel K. L. Baum M. J. (2009). Adult testosterone treatment but not surgical disruption of vomeronasal function augments male-typical sexual behavior in female mice. J. Neurosci. 29, 7658–7666. 10.1523/JNEUROSCI.1311-09.2009 19535577 PMC2717508

[B40] Maruniak J. A. Wysocki C. J. Taylor J. A. (1986). Mediation of male mouse urine marking and aggression by the vomeronasal organ. Physiol. Behav. 37, 655–657. 10.1016/0031-9384(86)90300-8 3749330

[B41] Matsunami H. Buck L. B. (1997). A multigene family encoding a diverse array of putative pheromone receptors in mammals. Cell 90, 775–784. 10.1016/S0092-8674(00)80537-1 9288756

[B42] Menco B. P. Carr V. M. Ezeh P. I. Liman E. R. Yankova M. P. (2001). Ultrastructural localization of G-proteins and the channel protein TRP2 to microvilli of rat vomeronasal receptor cells. J. Comp. Neurol. 438, 468–489. 10.1002/cne.1329 11559902

[B43] Meredith M. (1986). Vomeronasal organ removal before sexual experience impairs male hamster mating behavior. Physiol. Behav. 36, 737–743. 10.1016/0031-9384(86)90362-8 3714848

[B44] Meredith M. (1998). Vomeronasal, olfactory, hormonal convergence in the brain. Cooperation or coincidence? Ann. N.Y. Acad. Sci. 855, 349–361. 10.1111/j.1749-6632.1998.tb10593.x 9929627

[B45] Norlin E. M. Gussing F. Berghard A. (2003). Vomeronasal phenotype and behavioral alterations in G alpha i2 mutant mice. Curr. Biol. 13, 1214–1219. 10.1016/S0960-9822(03)00452-4 12867032

[B46] Oboti L. Perez-Gomez A. Keller M. Jacobi E. Birnbaumer L. Leinders-Zufall T.. (2014). A wide range of pheromone-stimulated sexual and reproductive behaviors in female mice depend on G protein Galphao. BMC Biol. 12:31. 10.1186/1741-7007-12-31 24886577 PMC4038847

[B47] Omura M. Mombaerts P. (2014). Trpc2-expressing sensory neurons in the main olfactory epithelium of the mouse. Cell Rep. 8, 583–595. 10.1016/j.celrep.2014.06.010 25001287

[B48] Omura M. Mombaerts P. (2015). Trpc2-expressing sensory neurons in the mouse main olfactory epithelium of type B express the soluble guanylate cyclase Gucy1b2. Mol. Cell. Neurosci. 65, 114–124. 10.1016/j.mcn.2015.02.012 25701815 PMC4396857

[B49] Pankevich D. E. Baum M. J. Cherry J. A. (2004). Olfactory sex discrimination persists, whereas the preference for urinary odorants from estrous females disappears in male mice after vomeronasal organ removal. J. Neurosci. 24, 9451–9457. 10.1523/JNEUROSCI.2376-04.2004 15496681 PMC6730103

[B50] Pankevich D. E. Cherry J. A. Baum M. J. (2006). Effect of vomeronasal organ removal from male mice on their preference for and neural Fos responses to female urinary odors. Behav. Neurosci. 120, 925–936. 10.1037/0735-7044.120.4.925 16893298 PMC2263134

[B51] Papes F. Logan D. W. Stowers L. (2010). The vomeronasal organ mediates interspecies defensive behaviors through detection of protein pheromone homologs. Cell 141, 692–703. 10.1016/j.cell.2010.03.037 20478258 PMC2873972

[B52] Paup D. C. Coniglio L. P. Clemens L. G. (1972). Masculinization of the female golden hamster by neonatal treatment with androgen or estrogen. Horm. Behav. 3, 123–131. 10.1016/0018-506X(72)90014-1 4681738

[B53] Pfeiffer C. A. Johnston R. E. (1994). Hormonal and behavioral responses of male hamsters to females and female odors: roles of olfaction, the vomeronasal system, and sexual experience. Physiol. Behav. 55, 129–138. 10.1016/0031-9384(94)90020-5 8140156

[B54] Powers J. B. Winans S. S. (1975). Vomeronasal organ: critical role in mediating sexual behavior of the male hamster. Science 187, 961–963. 10.1126/science.1145182 1145182

[B55] Ryba N. J. Tirindelli R. (1997). A new multigene family of putative pheromone receptors. Neuron 19, 371–379. 10.1016/S0896-6273(00)80946-0 9292726

[B56] Saito T. R. Moltz H. (1986). Copulatory behavior of sexually naive and sexually experienced male rats following removal of the vomeronasal organ. Physiol. Behav. 37, 507–510. 10.1016/0031-9384(86)90215-5 3749310

[B57] Schwanzel-Fukuda M. (1999). Origin and migration of luteinizing hormone-releasing hormone neurons in mammals. Microsc. Res. Tech. 44, 2–10. 9915559 10.1002/(SICI)1097-0029(19990101)44:1<2::AID-JEMT2>3.0.CO;2-4

[B58] Spehr J. Hagendorf S. Weiss J. Spehr M. Leinders-Zufall T. Zufall F. (2009). Ca2+ -calmodulin feedback mediates sensory adaptation and inhibits pheromone-sensitive ion channels in the vomeronasal organ. J. Neurosci. 29, 2125–2135. 10.1523/JNEUROSCI.5416-08.2009 19228965 PMC6666346

[B59] Stowers L. Holy T. E. Meister M. Dulac C. Koentges G. (2002). Loss of sex discrimination and male-male aggression in mice deficient for TRP2. Science 295, 1493–1500. 10.1126/science.1069259 11823606

[B60] Thor D. H. Flannelly K. J. (1977). Peripheral anosmia and social investigatory behavior of the male rat. Behav. Biol. 20, 128–134. 10.1016/S0091-6773(77)90682-4 869849

[B61] Tinbergen N. (1989). The Study of Instinct (Oxford; New York: Clarendon Press; Oxford University Press).

[B62] Tirindelli R. Dibattista M. Pifferi S. Menini A. (2009). From pheromones to behavior. Physiol. Rev. 89, 921–956. 10.1152/physrev.00037.2008 19584317

[B63] Vandenbergh J. G. (1983). Pheromones and Reproduction in Mammals (New York, NY: Academic Press).

[B64] Winans S. S. Powers J. B. (1977). Olfactory and vomeronasal deafferentation of male hamsters: histological and behavioral analyses. Brain Res. 126, 325–344. 10.1016/0006-8993(77)90729-6 861723

[B65] Wu M. V. Manoli D. S. Fraser E. J. Coats J. K. Tollkuhn J. Honda S.. (2009). Estrogen masculinizes neural pathways and sex-specific behaviors. Cell 139, 61–72. 10.1016/j.cell.2009.07.036 19804754 PMC2851224

[B66] Wysocki C. J. Lepri J. J. (1991). Consequences of removing the vomeronasal organ. J. Steroid Biochem. Mol. Biol. 39, 661–669. 10.1016/0960-0760(91)90265-7 1892795

[B67] Wysocki C. J. Yamazaki K. Curran M. Wysocki L. M. Beauchamp G. K. (2004). Mice (*Mus musculus*) lacking a vomeronasal organ can discriminate MHC-determined odortypes. Horm. Behav. 46, 241–246. 10.1016/j.yhbeh.2004.02.010 15325225

[B68] Yang C. Delay R. J. (2010). Calcium-activated chloride current amplifies the response to urine in mouse vomeronasal sensory neurons. J. Gen. Physiol. 135, 3–13. 10.1085/jgp.200910265 20038523 PMC2806418

[B69] Zhang P. Yang C. Delay R. J. (2008). Urine stimulation activates BK channels in mouse vomeronasal neurons. J. Neurophysiol. 100, 1824–1834. 10.1152/jn.90555.2008 18701755 PMC2576191

[B70] Zhang P. Yang C. Delay R. J. (2010). Odors activate dual pathways, a TRPC2 and a AA-dependent pathway, in mouse vomeronasal neurons. Am. J. Physiol. Cell Physiol. 298, C1253–C1264. 10.1152/ajpcell.00271.2009 20147653 PMC2867386

[B71] Zufall F. (2014). TRPs in olfaction. Handb. Exp. Pharmacol. 223, 917–933. 10.1007/978-3-319-05161-1_8 24961974

